# Experimental model for controlled endoscopic subepithelial vocal fold injury in rats

**DOI:** 10.1590/acb370106

**Published:** 2022-04-08

**Authors:** Laszlo Peter Ujvary, Cristina Maria Blebea, Maximilian George Dindelegan, Cristina Tiple, Bogdan Sevastre, Alma Aurelia Maniu, Magdalena Chirilă, Marcel Cosgarea

**Affiliations:** 1 Assistant Professor. University of Medicine and Pharmacy “Iuliu Hatieganu” –Department of Otolaryngology – Cluj-Napoca, Romania.; 2 Fellow PhD degree. University of Medicine and Pharmacy “Iuliu Hatieganu” – Department of Otolaryngology – Cluj-Napoca, Romania.; 3 Associate Professor. University of Agricultural Sciences and Veterinary Medicine – Department of Pathophysiology – Cluj-Napoca, Romania.; 4 Associate Professor. University of Medicine and Pharmacy “Iuliu Hatieganu” – Department of Otolaryngology – Cluj-Napoca, Romania.; 5 Professor. University of Medicine and Pharmacy “Iuliu Hatieganu” – Department of Otolaryngology – Cluj-Napoca, Romania.

**Keywords:** Vocal Cords, Endoscopy, Models, Animal, Rats

## Abstract

**Purpose:**

To present a detailed, reproducible, cost-efficient surgical model for controlled subepithelial endoscopic vocal fold injury in the rat model.

**Methods:**

Six male Sprague Dawley rats were enrolled in the experiment. The left vocal folds were used to carry out the injury model, and the right vocal fold served as control. After deep sedation, the rats were placed on a custom operating platform. The vocal fold injury by subepithelial stripping was carried out using custom-made microsurgical instruments under endoscopic guidance. Data were analyzed for procedural time and post-procedural pain. Microcomputed tomography (micro-CT) scan and histologic images were obtained to assess the length, area, and depth of injury to the vocal fold.

**Results:**

The mean procedural time was 112 s. The mean control vocal fold length was 0.96 ± 0.04 mm. The mean vocal fold injury length was 0.53 ± 0.04 mm. The mean vocal fold surface was 0.18 ± 0.01 mm^2^ with a mean lesion area of 0.05 ± 0.00 mm^2^. Mean vocal fold injury depth was 375.4 ± 42.8 μm. The lesion length to vocal fold length ratio was 0.55 ± 0.03, as well as lesion area to vocal fold surface area was 0.29 ± 0.02.

**Conclusions:**

Our described experimental vocal fold injury model in rats is found to be fast, safe, cost-efficient, and reproducible with a rapid learning curve.

## Introduction

Experimental models are essential in consolidating fundamental principles and scaling them to clinical research and application. For this purpose, the use of animal models addresses various objective scientific questions. While no animal model provides an ideal representation of the human vocal fold, different species hold unique advantages for particular experimental applications^1^^,^ 2.

The rat model holds the advantage of being used in all research areas and already presents a vast majority of research data available. Although technically challenging to perform standardized lesions on the rat vocal fold, it holds the advantage of presenting the same three-layered vocal fold structure as humans and having similar fibrous protein composition of the extracellular matrix (ECM)^3^.

The lamina propria exposed due to an injury of the vocal fold epithelium will lead to modifications in the composition of the ECM. These modifications will impair the vibratory function of the vocal fold, which clinically will manifest as dysphonia^4^^-^ 6.

Experimental models up to date have investigated vocal fold remodeling, repair and gave chronological insight to vocal fold healing following surgical injury by describing structural and functional modifications of the lamina propria and epithelium^7^^,^ 8.

In recent years, clinical need drove experimental medicine further by researching new methods to promote healing and modulate scarring response following acute vocal fold injuries. Regenerative therapy and cell-based therapy, using mesenchymal stem cells, platelet-rich plasma, fibroblasts, and other proliferative agents or growth factors, have been studied using dogs, pigs, rats, rabbits, and mice with promising results^9^^-^ 13.

Regardless of the scope of each study, what all models share is a methodology for controlled vocal fold injury. However, the majority of authors describe the injury process as vocal fold stripping. From the methodological descriptions in the rat model, stripping is understood as injuring the deep layer of the lamina propria and exposing the thyroarytenoid muscle.

Patients undergoing phonosurgery would greatly benefit from regenerative therapies to reduce voice rest required after surgery and eliminate possible scarring of the vocal folds. An experimental subepithelial injury model would be required to study the effects of diverse bioactive agents to promote wound healing and reduce vocal fold scarring.

A classification method for rat vocal fold injuries, proposed by Imaizumi *et al*.^14^, describes three types of vocal fold injuries that correspond to the classification proposed by the European Laryngological Society so that experimental studies would be more relevant for clinical application. Considering these classifications, a vocal fold stripping in the rat model as described in the literature would correspond to a type II cordectomy in humans. Phonosurgery for benign vocal fold lesions (type I cordectomy) would be best described as subepithelial vocal fold injuries in the rat model (Imaizumi classification)^14^^,^ 15.

To the best of our knowledge, there is no current experimental surgical model for subepithelial vocal fold lesions in a rat model; thus, the purpose of this study is to describe an experimental model to fit this purpose.

## Methods

## Study type

Prospective, comparative, experimental animal study.

## Study design

Two groups were established. Each vocal fold from each rat was assigned as follows: vocal fold injury group: left vocal fold (n = 6); control group: right vocal fold (n = 6).

## Study group characteristics

Six male Sprague Dawley rats (393 g ± 27.96 g) were used in the study. The rats were housed in separate cages, with constant temperature (23 ± 2 °C). Ad libitum feeding and 12-h day and night cycles were provided. No feeding was performed 12 h before the experiment. All six rats underwent unilateral subepithelial vocal fold injury (type I cordectomy). Surgery time and postprocedural pain intensity were noted. All procedures were reviewed and approved by the Ethics Committee (68/12.06.2017) and followed the Animal Research: Reporting of In Vivo Experiments (ARRIVE) guidelines recommendations.

## Anesthesia

Initially, induction anesthesia with Isoflurane (2–3% with a flow of 1l/min) was administered^8^. Appropriate analgesia and sedation were obtained using 9 mg/kg xylazine and 90 mg/kg of ketamine, administered intramuscularly. An additional 0.1 mL, 2 mg/kg of topical lidocaine was applied to the epiglottis and vocal folds to limit involuntary movement with a micropipette.

## Animal positioning, visualization, and instrumentation

**Figure 1:**
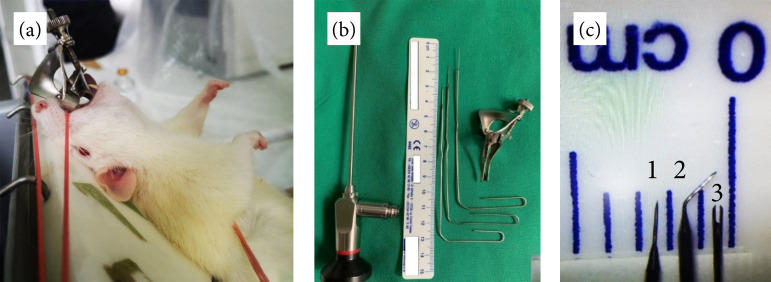
Rat positioning, equipment, and setup. **(a)** Custom-made operating platform, with the rat positioned semi vertically and secured with three rubber bands at the level of the rare, front limbs and incisive teeth. **(b)** Equipment consisting of 2.7 mm-diameter 30° endoscope, custom electron microscopy instruments for lesion induction and a repurposed Voltolini self-retractor nasal speculum. The speculum blade margins were narrowed and straightened so that they fit through the rat’s mouth to aid in the pharyngeal lumen patency. **(c)** Close up view of the instruments used for vocal fold injury (1) 0.12 mm microneedle; (2) 0.5 mm microspatula (3) 0.25 mm microfork.

**Figure 2:**
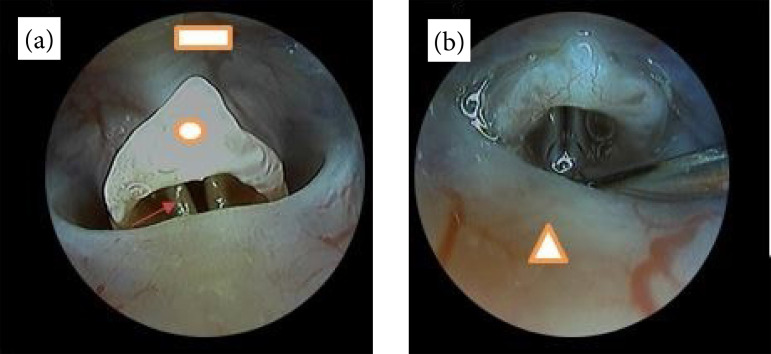
Endoscopic images of the rat hypopharynx and larynx. **(a)** In posterior decubitus in a nearly horizontal position, the vallecula is visible in the superior aspect of the image (*square*), followed by the epiglottis (*circle*). The arytenoids (*arrow*) can be easily mistaken for the true vocal folds. The true vocal folds lie deep to the base of the epiglottis and visualization can become difficult without appropriate anesthesia. This is due to the reflex movement of the epiglottis. **(b)** The posterior fold of mucosa (*triangle*) represents the soft palate. In the supraglottic region, sputum may be visible and may impair visibility.

The operation platform was made from a custom support system (Fig. 1). The tongue of the rat was pulled outward to elevate the base and lift the epiglottis. For securing the pharyngeal lumen patency, we repurposed a Voltolini (Duplay) self-retractor speculum to act as a suspension laryngoscope (Fig. 1). The blades of the speculum were straightened and the width was ground so it could comfortably fit between the base of the tongue and the posterior pharyngeal wall. As the thumbscrew was tightened, the two blades widened the pharyngeal lumen to ease visibility of the glottis. Sputum, if needed, was aspirated from the laryngeal lumen with no. 5 Frasier suction tip attached to a surgical aspirator, set to minimum power. Endoscopic laryngeal visualization and image acquisition were obtained using a 30°, 2.7-mm diameter rigid endoscope, equipped with a portable light source and a Firefly Pro wireless camera (Fig. 2). The subepithelial vocal fold lesions were carried out using custom-made microsurgical instruments from repurposed electron microscopy instruments. The instruments’ tip was cut and laser welded to a steel handle to provide additional length and rigidity (Fig. 1).

## Pain assessment

After completing the experiment, the rats were placed in separate cages and closely monitored for 4 hours in 15 min increments. Pain assessment using the Rat Grimace Scale (RGS) was noted^16^.

## Microcomputed tomography (micro-CT) scan and histologic analysis

**Figure 3:**
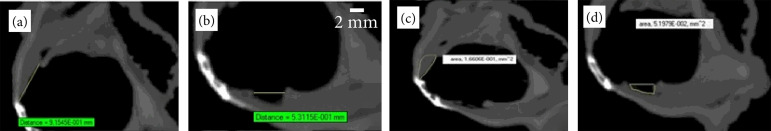
Micro-CT measurements of the rat vocal fold parameters. **(a)** Micro-CT measurement of normal vocal fold length, measured from the vocal process of the arytenoid to the attachment of the vocal fold to the thyroid cartilage. **(b)** Length of controlled endoscopic vocal fold injury, measured from the anterior to the posterior edge of the lack of substance. **(c)** Surface area of normal rat vocal fold measured form free margin of vocal fold-medial; vocal process of arytenoid-posterior; medial lamina of the thyroid cartilage-lateral; anterior attachment of the vocal fold-anterior. **(d)** Surface area of vocal fold lesion measured from the deepest point-lateral; posterior edge of lesion-posterior; anterior edge of lesion- anterior; virtual line bridging the anterior and posterior epithelial medial surface.

**Figure 4:**
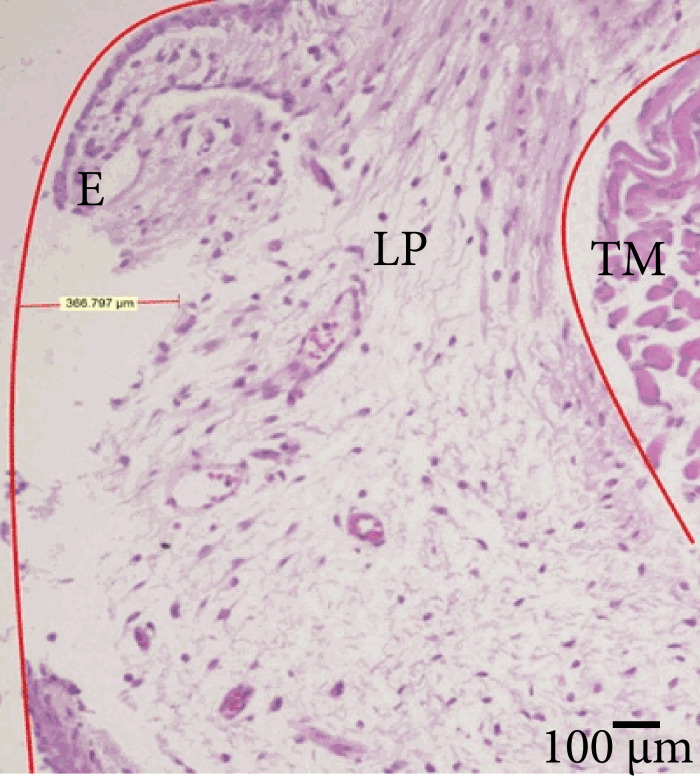
Histologic confirmation of lesion depth. Microscopic examination of the vocal fold (200× magnification) shows stratified nonsquamous keratinized epithelium with discontinuation. The superficial layer of the lamina propria presents edema, capillary congestion with few lymphocytes and plasmocytes. No exposure of the thyroarytenoid muscle is visible. The vocal ligament is intact. E = epithelium; LP = lamina propria; TM = thyroarytenoid muscle.

All subjects were euthanized with isoflurane (6%) overdose until breathing and heartbeat stopped. Euthanasia was confirmed with cervical dislocation. All euthanasia procedures followed national guidelines for laboratory animals. The larynx of all six rats was harvested and prepared in 10% formaldehyde for micro-CT and histological examination. After a 24-h fixation, micro-CT images (Bruker Skyscan Belgium Model 1172) were obtained and processed using intermediate resolution with a slice thickness of 13.6 μm. Vocal fold and injury assessment by micro-CT was made by measuring total vocal fold length, subepithelial injury length (between the edges of the detached mucosa), vocal fold surface area, and injury surface area (lack of substance in the vocal fold). Three independent operators repeated micro-CT measurements two times for each parameter. The final value was considered the average of all measurements (Fig. 3). Histologic hematoxylin & eosin (H&E) staining was done on all specimens to assess the depth of the lesion. The lesion depth was determined by measuring the lack of substance from the epithelial lining to the most profound point of injury. Two independent operators performed measurements two times, and the average of these findings was considered as the final value (Fig. 4). The ratio between vocal fold length to lesion length and vocal fold surface area to lack of substance area were also evaluated to assess the constancy of the lesions.

## Data analysis

Data was presented as mean ± standard deviation for values representing injury length, area, and depth, and the ratio between injury length to vocal fold length as well as lack of substance to vocal fold surface area.

## Results

**Figure 5:**
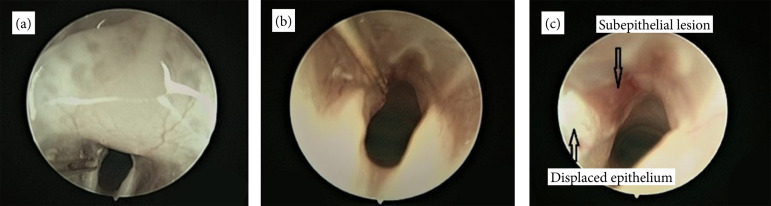
Subepithelial lesion induction. **(a)** The microfork and microneedle were used interchangeably to injure the vocal fold and detach it anteriorly. **(b)** Insertion of the microneedle in the subepithelial space. **(c)** The microspatula was used to scrape the vocal fold epithelium and displace it posteriorly, leaving the profound layer of the lamina propria intact.

All six animals submitted to the vocal fold stripping procedure survived. The surgical setup and anesthesia protocol permitted successful visualization and vocal fold injury execution on all six rats. Endoscopic image representation for workflow documentation is indicated in Figs. 2 and 5.

Procedure time, recovery time, and RGS score for postoperative pain are shown in Table 1. The procedural time shortened after the initial vocal fold injury. The average time from lesion induction was 112 s. Recovery time was between 30–45 min for four of the rats and between 45–60 min for two of the rats.

**Table 1:** Procedure characterization regarding executionspeed, recovery time and postoperative pain.

Nr.	Procedure time (s)	Recovery time (min)	RGS
1	170	30–45	0
2	96	30–45	0
3	103	45–60	0
4	86	30–45	0
5	111	30–45	0
6	107	45–60	0

RGS (Rat Grimace Scale): 0 = no pain; 1 = moderate pain; 2 = intense pain; consideringseparate score for orbital tightening, cheek flattening, ear changes, whisker changes.

Cumulated data is represented in Table 2. The lesion length (LL) to vocal fold length (VFL) ratio was 0.55 ± 0.03 as well as lesion area (LA) to vocal fold surface area (VFSA) was 0.29 ± 0.02. Both ratio values were constant with minor deviations from the mean.

**Table 2:** Micro-CT and histological measurements for vocal fold parameters in all subjects.

Control group n=6				Injury group n=6					
Nr.	VFL (mm)	VFSA (mm^2^)		Nr.	LL (mm)	LA (mm^2^)	LL/ VFL	LA/VFSA	LD (μm)
1.	0.93	0.17		1.	0.52	0.05	0.56	0.29	332.72
2.	1.03	0.19		2.	0.60	0.05	0.58	0.26	431.14
3.	0.96	0.18		3.	0.56	0.06	0.58	0.33	366.77
4.	0.92	0.17		4.	0.53	0.05	0.58	0.29	398.31
5.	0.95	0.17		5.	0.48	0.05	0.51	0.29	402.62
6.	0.96	0.18		6.	0.50	0.05	0.52	0.28	321.57
Mean ± SD	0.96 ± 0.04	0.17 ± 0.01		Mean ± SD	0.53 ± 0.04	0.05±0.00	0.55 ± 0.03	0.29 ± 0.02	375.43 ± 42.88

Vocal fold length - VFL; Vocal fold surface area – VLSA; Lesion length – LL; Lesion area (lack of substance) – LA; Lesion depth – LD; standard deviation – SD

## Discussion

The goal of our study was to create an affordable and replicable vocal fold injury model for subepithelial lesions that could be used to simulate human type I cordectomies in the rat model. During the process, we created a custom operating platform, repurposed a Voltolini (Duplay) self-retracting nasal speculum to serve as a suspension laryngoscope, and adapted custom surgical instruments for the purpose. We used a 30° 2.7-mm diameter endoscope for visualization due to its availability and affordability compared to smaller diameter endoscopes. The 2.7-mm diameter endoscope is well fitted when compared to the endoluminal space. The placement of the surgical instruments besides the endoscope did not pose problems in execution.

To reduce the sputum in the laryngeal lumen, we used a surgical aspirator in two cases. The aspirator was attached to a no. 5 Frasier suction tip. We encountered no difficulty in the procedure and avoided the use of extra medication. Care must be taken not to touch the vocal folds during aspiration because of potential suction pressure and edema.

The vocal fold injury was created using custom electron microscopy instruments due to the fine tip and small size. The microfork instrument and the microspatula were used in all cases. The microneedle was used when the anterior epithelial attachment was harder to displace. A 22G-26G needle, as described in the literature, would have made it difficult to execute the subepithelial injury given that the vocal fold presents only a 0.2 mm thickness^17^. Our controlled endoscopic surgical vocal fold injuries had an average length of 0.53 ± 0.04 mm. This would be appropriate to simulate repeated subepithelial vocal fold injuries in a standardized fashion.

When placing the microfork instrument on the superior surface of the vocal fold, it is important to take into account the movement of the vocal fold and not to injure the underlying thyroarytenoid muscle or the vocal ligament with the stabbing motion. We recommend placing only one point of the fork on the vocal fold surface so that the other point left in the glottic space would act as counter support, limiting the depth of the lesion. We used anterior and posterior swinging motions to separate the epithelium. If the epithelium was not separated, the microneedle was used to separate the epithelium from its anterior attachment. To prevent injury to the deeper layers of the lamina propria, the microspatula was used to displace the epithelium posteriorly. Our lesion surface area (lack of substance) was constant, averaging 0.05 ± 0.00 mm^2^. These results indicate a constant placement of the instruments on the vocal fold surface for inducing the lesion.

Our measured vocal fold lengths were constant with a mean of 0.96 ± 0.04 mm. The findings are slightly lower than the 1 mm length described in the literature. This difference may be determined by the different measuring methods used (histology vs. micro-CT). In micro-CT imaging, it is more difficult to accurately distinguish tissue variations solely on density. This makes it harder to precisely observe the transition from the posterior aspect of the true vocal fold to the arytenoid mucosa.

The ratio values regarding injury length to vocal fold length indicate that by using this methodology, we were able to reproduce even injuries with minor deviations from the mean, and the vocal fold injury is constant compared to the vocal fold length.

Although we secured intraoperative confirmation of the lesion depth, micro-CT images do not allow distinguishing the density of the thyroarytenoid muscle, lamina propria, and epithelium. To address this shortcoming, we used H&E staining to confirm the depth of the injury and ensure that all lesions were subepithelial and did not injure the vocal ligament or the thyroarytenoid muscle. The mean lesion depth was 375.4 ± 42.88 μm and was superficial to the vocal ligament.

Although there is a vast literature on vocal fold studies using the rat model, the number of original detailed surgical methodologies is limited. To the best of our knowledge, the first similar experimental model using cold instruments was described by Tateya *et al*.^8^. Other workgroups adhered to this model, reproducing it integrally or making minor adjustments to suit the scope of the study. These authors pioneered the rat vocal fold surgical model. They described the use of a custom operating platform, a custom steel wire laryngoscope, and a 1.9 mm 25° endoscope for vocal fold visualization. The vocal fold injury was made with a 26-gauge needle and microforceps.

As far as we are aware, in the last decade, no other article focused on the detailed technical description of a vocal fold injury in a rat model. To the best of our knowledge, this is the first study describing a subepithelial vocal fold injury in the rat model. The mouse surgical model was presented by Yamashita *et al*.^18^ by describing a surgical protocol for vocal fold lesions. They described it as a necessity due to the more extensive data available for targeted genetic manipulation in mice. The model would serve as guidance for controlled vocal fold injury in favor of using the data for further advances in vocal fold pathology.

Opposed to cold instruments, surgical lasers in the vocal fold injury model are somewhat limited. Only a few articles came to our attention using a nonsystematic search. Vocal fold scar modulating effects of the KTP laser was the main area of interest^19^^,^ 20, but recently, the use of the novel blue light laser shows better results compared to the KTP laser^21^.

When choosing the rat breed, we argue that the Sprague Dawley rats are better suited than Wistar rats because of their slightly larger size, weight, faster growth rate, and calmer personality. These traits would be helpful in animal manipulation as well as reducing the time of follow-up on desired outcomes due to faster growth rates^22^.

## Conclusion

We believe that our experimental endoscopic vocal fold injury model addresses the problem of cost efficiency, reproducibility, and procedural time. Endoscopes are an excellent way to visualize and control the injury length and depth. Our results come as a completion to the literature intending to offer a further outlook on the complex nature of vocal fold healing and dysphonia.
